# Biodistribution of Mesenchymal Stromal Cells Labeled with [^89^Zr]Zr-Oxine in Local Radiation Injuries in Laboratory Animals

**DOI:** 10.3390/molecules28207169

**Published:** 2023-10-19

**Authors:** Tatiana A. Astrelina, Vitaliy A. Brunchukov, Galina E. Kodina, Viktor B. Bubenshchikov, Anton A. Larenkov, Aleksandr S. Lunev, Kristina A. Petrosova, Anna A. Rastorgueva, Irina V. Kobzeva, Daria Y. Usupzhanova, Victoria A. Nikitina, Kristina A. Malsagova, Ludmila I. Kulikova, Alexander S. Samoilov, Vasiliy I. Pustovoyt

**Affiliations:** 1 State Research Center—Burnasyan Federal Medical Biophysical Center of Federal Medical Biological Agency, 123182 Moscow, Russia; t_astrelina@mail.ru (T.A.A.); brunya2008@yandex.ru (V.A.B.); gkodina@yandex.ru (G.E.K.); bubenschikov2011@yandex.ru (V.B.B.); anton.larenkov@gmail.com (A.A.L.); mr.alekslunev@gmail.com (A.S.L.); christfmbc@gmail.com (K.A.P.); rastorgueva.ann@gmail.com (A.A.R.); irina-kobzeva@yandex.ru (I.V.K.); usupzhanova94@mail.ru (D.Y.U.); nikitinava@yandex.ru (V.A.N.); asamoilov@fmbcfmba.ru (A.S.S.); vipust@yandex.ru (V.I.P.); 2 Institute of Biomedical Chemistry, Biobanking Group, 119121 Moscow, Russia; likulikova@mail.ru; 3 Institute of Mathematical Problems of Biology RAS—The Branch of Keldysh Institute of Applied Mathematics of Russian Academy of Sciences, 142290 Pushchino, Russia; 4 Institute of Theoretical and Experimental Biophysics, Russian Academy of Sciences, 119991 Pushchino, Russia

**Keywords:** mesenchymal stromal cells, local radiation injury, positron emission tomography, [^89^Zr]Zr-oxine

## Abstract

Background: Tracking the migration pathways of living cells after their introduction into a patient’s body is a topical issue in the field of cell therapy. Questions related to studying the possibility of long-term intravital biodistribution of mesenchymal stromal cells in the body currently remain open. Methods: Forty-nine laboratory animals were used in the study. Modeling of local radiation injuries was carried out, and the dynamics of the distribution of mesenchymal stromal cells labeled with [^89^Zr]Zr-oxine in the rat body were studied. Results: the obtained results of the labelled cell distribution allow us to assume that this procedure could be useful for visualization of local radiation injury using positron emission tomography. However, further research is needed to confirm this assumption. Conclusions: intravenous injection leads to the initial accumulation of cells in the lungs and their subsequent redistribution to the liver, spleen, and kidneys. When locally injected into tissues, mesenchymal stromal cells are not distributed systemically in significant quantities.

## 1. Introduction

With ever-growing interest in cell therapy, there remains the need for long-term tracking of the migration pathways of living cells after their introduction into a patient’s body. Tracking of individual cell types in the body (e.g., eosinophils and neutrophils [1,2], T lymphocytes [3,4,5], and dendritic cells [6]) is assessed using *in vivo* imaging techniques. Currently, monitoring the migration of transplanted or intravenously injected cells requires a biopsy procedure to be performed, making it difficult to assess *in vivo* (*in vivo*/“online”) the effects of cell modification or their administration route for enhancing cell migration to target organs. The most suitable methods both for studying cell migration and for clinical applications include visualization of cells labeled with various radionuclides using single-photon emission computed tomography (SPECT) or positron emission tomography (PET). Labeling using various lipophilic complexes with gamma- or positron-emitting radionuclides is the method used most commonly for this purpose. Investigation of the migration pathways of autologous leukocytes labeled with radionuclides in the body using scintigraphy or SPECT is a widely used technique that has been employed in nuclear medicine for over 30 years [7]. Most frequently, this method is used in clinical practice to identify infectious or aseptic inflammation areas [8,9]. The standard procedure for labeling leukocytes is based on the mechanism of nonspecific accumulation of lipophilic complexes of indium-111 with 8-hydroxyquinoline (oxine) [10], tropolone [11], and some bidentate chelating agents [12]. Later, similar preparations were obtained with technetium-99m [13]. However, the nuclear physical characteristics of indium-111 are not optimal for obtaining clear visualization in SPECT, and the half-life of technetium-99m is 6 h, which does not allow one to perform studies delayed by more than a day.

PET has a higher resolution than SPECT. When using a positron-emitting radionuclide with a half-life suitable for research purposes, PET will have undoubted advantages.

The long-lived positron-emitting radionuclide zirconium-89 is the most suitable for reducing radiation exposure while maintaining a high sensitivity, resolution, and specificity sufficient for monitoring cell migration over several days. Zirconium-89 has a low maximum energy emitted by positrons, thus enabling high-resolution PET imaging. Its half-life of 3.27 days makes it possible to obtain a series of PET images over several days, allowing the relatively slowly occurring changes in distribution of labeled molecules or cells to be visualized. Like for indium-111, the complex of zirconium-89 with 8-hydroxyquinoline can be obtained through direct labeling. There has been a limited number of *in vitro* and laboratory animal studies that have shown the significance of [⁸⁹Zr]Zr-oxine for monitoring cell therapy in mouse models, including T cells [14,15], dendritic cells, NK cells, and bone marrow cells [16,17]. According to the authors of these studies, [⁸⁹Zr]Zr-oxine is a promising imaging agent for long-term monitoring of the migration pathways of various transplanted cells. Radioactively labeled cells introduced into the body can be visualized with a very high ratio between the radioactivities in the accumulation area and the background using PET.

The questions related to studying the possibility of long-term intravital biodistribution of mesenchymal stromal cells (MSCs) in the body remain open today. Treatment of local radiation injuries (LRIs) is challenging because of the metabolic and proliferative processes in tissues characteristic of radiation ulcers and changes in the state of both tissue and regional circulation in the damaged area. By assessing the volume of LRIs, one can identify the approach for its further treatment using systemic administration of MSCs and study the effectiveness of their accumulation (biodistribution) in the targets. Determination of the LRI volume and examination of the *in vivo* biodistribution of MSCs in the body is possible by labeling them with a radionuclide, which subsequently allows one to register ionizing radiation using direct radiometry or PET. This enables both visual and quantitative assessment of radiation accumulation. It is promising to use [⁸⁹Zr]Zr-oxine for intravital labeling of MSCs followed by PET imaging for this purpose. Total-body PET scanning after the introduction of cells labeled with zirconium-89 will significantly reduce the scanning time and the dose load both on isolated cells and the patient’s entire body [18].

The aim of this study was to investigate the *in vivo* biodistribution of [⁸⁹Zr]Zr-oxine-labeled mesenchymal stromal cells in a living organism through direct labeling using the pathway of passive diffusion through the cell membrane in local radiation injuries in laboratory animals.

## 2. Results

### 2.1. Immunological Characteristics and Viability of Mesenchymal Stromal Cells

**Figure 1:**
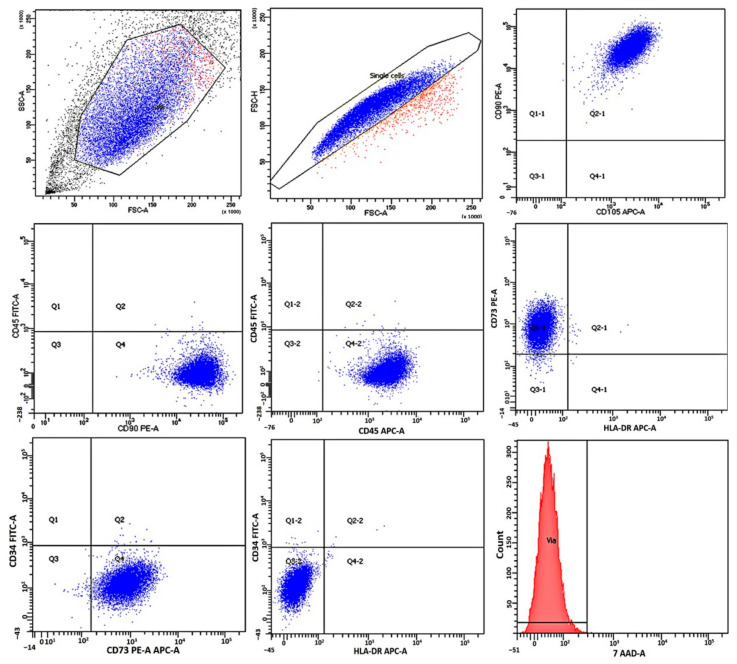
Immunophenotype of mesenchymal stromal cells from human gingival tissue (CD90+/CD105+73+/CD45-/CD34-/HLA-DR-, 7-ADD (99.5%). Analysis of human gingival mesenchymal rafter cells using flow cytometry for the expression of specific antigens in human gingival mesenchymal stromal cells. Graphs obtained as a result of flow cytometric analysis are presented. Mesenchymal truss cells of the human gingival mucosa of passages 3–5 (P4) were analyzed using flow cytometry after staining with fluorescein isothiocyanate (FITC), allophycocyanate (APC), or phycoerythrin (PE) and fixed on antibodies against the indicated proteins on the cell surface (blue color). The expression of the presented surface markers for MSCs was more than 90%.

Analysis of the MSC immunophenotype using flow cytometry revealed high expression of MSC markers (CD73, CD90, and CD105) in all the cell cultures; markers of hematopoietic and lymphocytic origin (CD34, CD45, and HLA-DR) were absent. The immunophenotype met the requirements posed by the International Organization for Cell Therapy of Human MSCs [19]. The MSCs retained a high proliferative activity and viability (98.21 ± 1.72% 7-ADD were negative) throughout the entire cultivation period (Figure 1). The details were previously published in ref. [19].

### 2.2. [^89^Zr]Zr-Oxine Synthesis, QC, and Cell Labeling Efficiency

**Figure 2:**
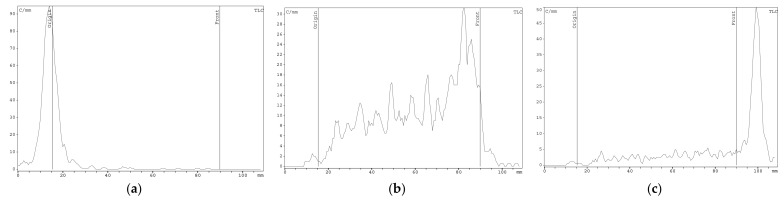
Examples of radio-TLC chromatograms of ^89^Zr preparations in the ethyl acetate/iTLC-SG system. (**a**) [^89^Zr]Zr-oxalate; (**b**) [^89^Zr]Zr-oxine (pH 7) after 1 h of incubation; (**c**) [^89^Zr]Zr-oxine (pH 7) after 24 h of incubation.

Typically, [^89^Zr]Zr-oxine is synthesized within the pH range of 7–8 [20,21]. In this study, the synthesis was carried out in the pH range of 7–9. According to the results of the TLC analysis, the reaction yield (radiochemical purity, RCP) at the given pH of the medium was 97.6 ± 1.8%. At the same time, the results of the TLC analysis often did not give a clear indication of the radiochemical purity of the preparations. The TLC system with ethyl acetate/iTLC-SG consistently did not provide complete separation of the components of the reaction mixture (Figure 2b). A similar case of incomplete separation in this system was reported previously [20]. The authors attributed the incomplete separation to dissociation of the complexes during the analysis. Clear relationships between chromatographic behavior and synthesis conditions were not found at this stage of the study. But, it was found that increasing the incubation time of the reaction mixture (up to 24 h) resulted in a clearer chromatographic pattern (Figure 2c). We assume that the incomplete separation may be due to the formation of intermediate [^89^Zr]Zr-oxine ([^89^Zr]Zr(oxine)_n_, n = 1–3) complexes and the kinetics of their mutual transformations. 

However, an alternative and more reliable method for analyzing radiochemical purity was needed. The liquid–liquid extraction (LLE) method (with chloroform and later with octanol-1) is recommended by European Pharmacopoeia to determine the RCP of the commonly used [^111^In]In-oxine [22]. This method (using chloroform) is applicable for Zr-oxine complexes [23,24].

**Figure 3:**
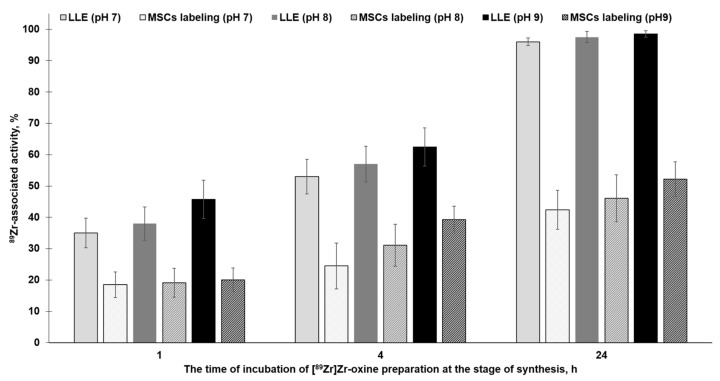
Dependence of the efficiency of [^89^Zr]Zr-oxine extraction using chloroform (RCP), as well as MSC cell labeling, on pH and incubation time of [^89^Zr]Zr-oxine preparations (mean ± SD, n = 5).

The extraction results showed that the value of radiochemical purity does not have a statistically significant difference between the samples prepared at pH 7–9. For the samples prepared by incubating the reaction mixture for 1 h at pH 7 and 9, the RCP was 36.5 ± 4.7% and 45.7 ± 7.1% (*p* > 0.05), respectively. But, at the same time, the radiochemical purity increased significantly with an increase in the incubation time of [^89^Zr]Zr-oxine samples: 40.1 ± 6.6 (1 h of incubation) vs. 97.3 ± 1.6 (24 h of incubation) (Figure 3).

The efficiency of cell labeling was determined using [^89^Zr]Zr-oxine samples prepared under various conditions—Figure 3. MSC cell labeling experiments showed no statistically significant difference in labeling efficiency using samples of [^89^Zr]Zr-oxine synthesized at different pH values. For example, in the case of 1 h incubated [^89^Zr]Zr-oxine samples, the labeling efficiency was 18.5 ± 6.6% (pH 7) vs. 20.1 ± 6.6% (pH 9), *p* > 0.05—Figure 3. But, at the same time, similar to the results of the extraction, the efficiency of cell labeling also increased with the use of [^89^Zr]Zr-oxine samples obtained during long-term incubation: 19.3 ± 3.7% (with 1 h incubated [^89^Zr]Zr-oxine preparations, pH 7–9) vs. 46.9 ± 7.1% (with 24 h incubated [^89^Zr]Zr-oxine preparation, pH 7–9), *p* > 0.05. Cell viability studies using the trypan blue staining assay have shown that 90–95% of living cells are retained in the cell suspension in all cases of [^89^Zr]Zr-oxine preparations. The study of the kinetics and peculiarities of the [^89^Zr]Zr-oxine complex formation, as well as optimization of its synthesis, are the subject of a separate study and is beyond the scope of the present work. For further studies on cell distribution *in vivo*, preparations of [^89^Zr]Zr-oxine obtained at pH 9 after incubation for 24 h were used (with RCP 98.5 ± 1.1% (LEE), giving a cell labeling efficiency of 52.2 ± 7.1%).

A noticeable efflux was observed for labeled MSCs in saline. The retention of ^89^Zr was 74 ± 3% after 24 h and 48 ± 4% after 7 days, regardless of the pH value at the synthesis stage. The results of the stability assessment are consistent with previously published data [19].

### 2.3. Study of the Distribution Dynamics of Mesenchymal Stromal Cells Labeled with [^89^Zr]Zr-Oxine in Animals on the Post-Irradiation Day

**Figure 4:**
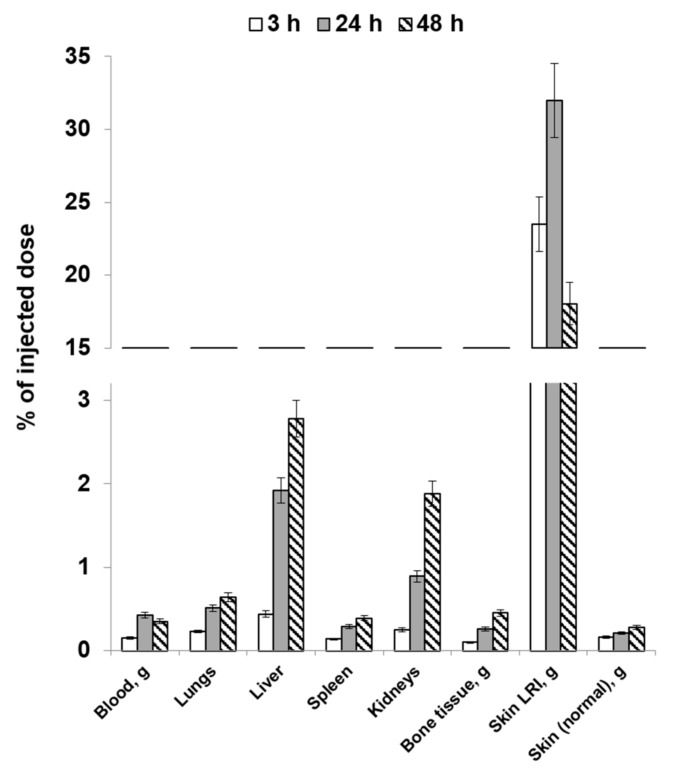
The average values of the distribution dynamics in the bodies of rats on post-irradiation day 1 (local injection).

**Figure 5:**
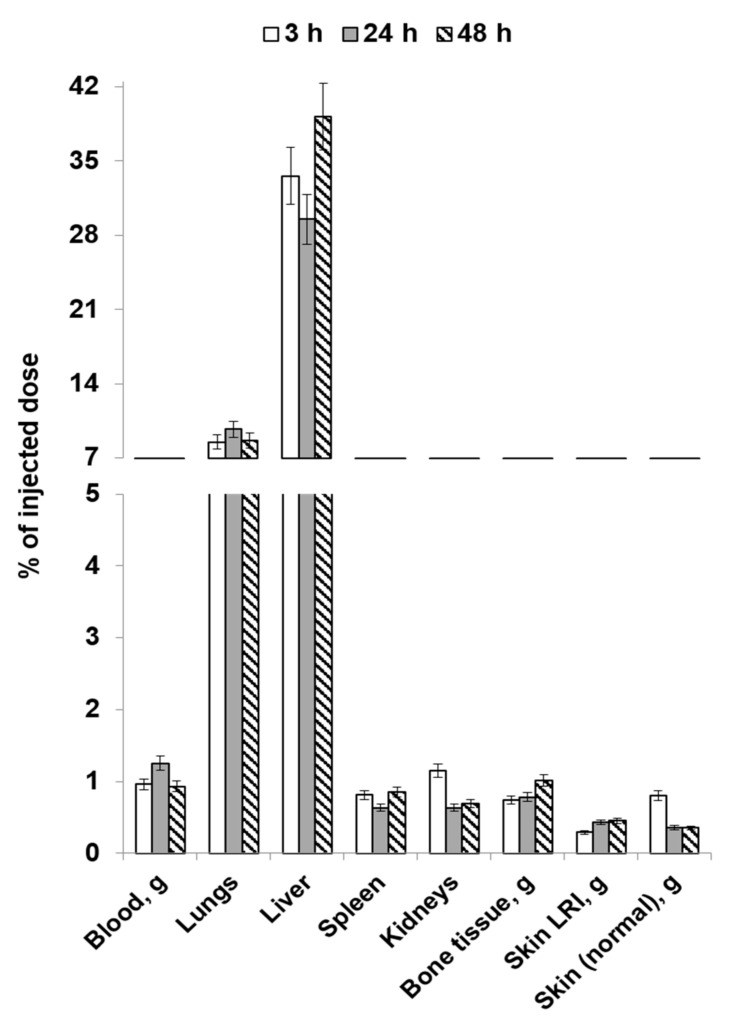
The average values of the distribution dynamics in the bodies of rats on post-irradiation day 1 (intravenous injection).

Figure 4 and Figure 5 list the average values of the obtained results of the study focusing on the distribution dynamics of [^89^Zr]Zr-oxine-labeled human gingival mucosal MSCs in the body of Wistar rats on day 1 post-irradiation through local and intravenous injection routes.

In the case of local injection, most of the injected cells remained in the pathological focus. Cell migration from the injection site was also observed; after 48 h, greater quantities of cells were accumulated in the liver compared to the other organs. Accumulation in the bone tissue, being characteristic of the biological behavior of unbound zirconium-89, was minimal at all the observation points. However, the observed rise in the accumulation of labeled cells indicates that the radionuclide release increases by study day 2. This phenomenon may demonstrate that some of the cells introduced into the body died. Radioactivity accumulation in the kidneys is a consequence of excretion of free zirconium-89 from the body.

Therefore, with a known lesion location, local administration of [^89^Zr]Zr-oxine-labeled MSCs will make it possible to visualize it against the background of intact skin using PET. In addition, having analyzed the resulting findings, we can assume that early treatment of acute radiation damage to skin can be performed through local administration of MSCs without significant cell population loss in non-target organs.

When administered intravenously, physiologically based accumulation was observed in the liver and kidneys, as well as in the lungs. Cell accumulation in the LRI was significantly lower 3 h after administration compared to the healthy skin area. However, activity accumulation in the LRI focus increased during the later period while declining in the intact skin. The resulting LRI/skin ratio values indicate that it is possible to visualize the radiation injury focus, although with insufficient contrast. This can be attributed to the early and insufficiently long period for developing an inflammatory response after animals’ exposure. Accordingly, the expected efficacy of cell therapy in such an early period after irradiation at the doses used will be insignificant. In addition, the data obtained indicate that a significant portion of radiolabeled MSCs administered intravenously will be absorbed by the liver and lungs without reaching the lesion. However, with an unspecified localization of the radiation injury focus, it is possible to use labeled MSCs capable of visualizing the injury focus and then conduct cell therapy using local injection.

### 2.4. Study of the Distribution Dynamics of Animals on Post-Irradiation Day 14

**Figure 6:**
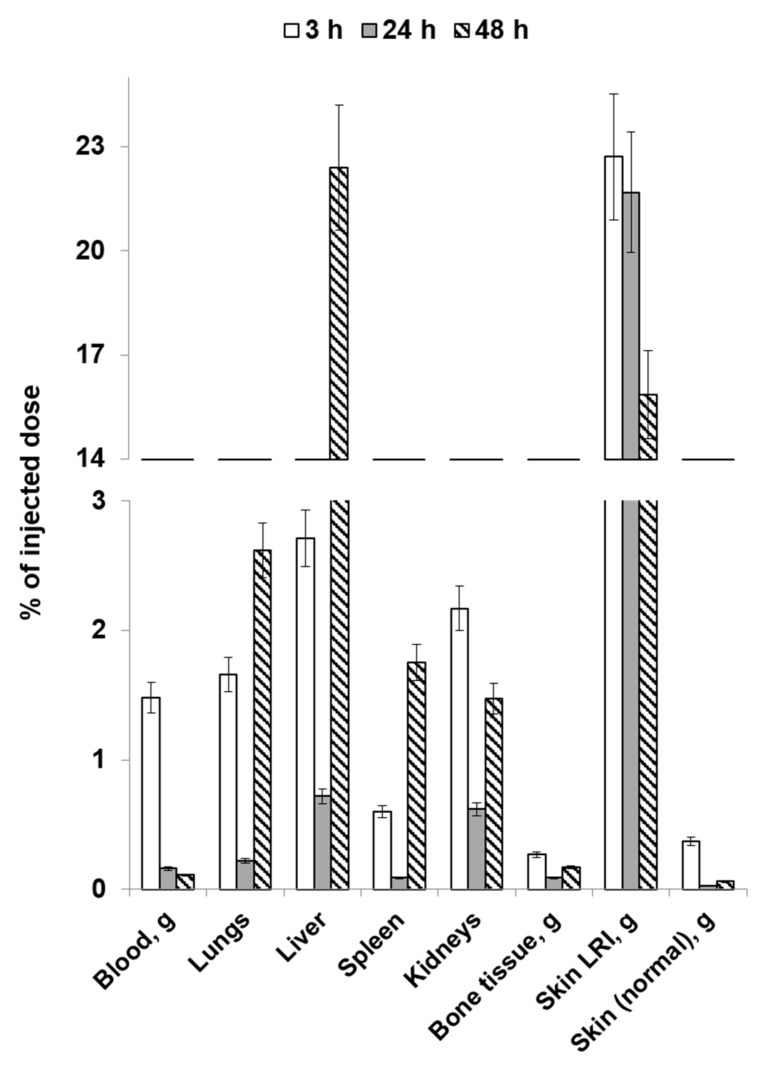
The average values of the distribution dynamics of [^89^Zr]Zr-oxine-labeled MSCs in the bodies of rats on post-irradiation day 14 (local injection).

**Figure 7:**
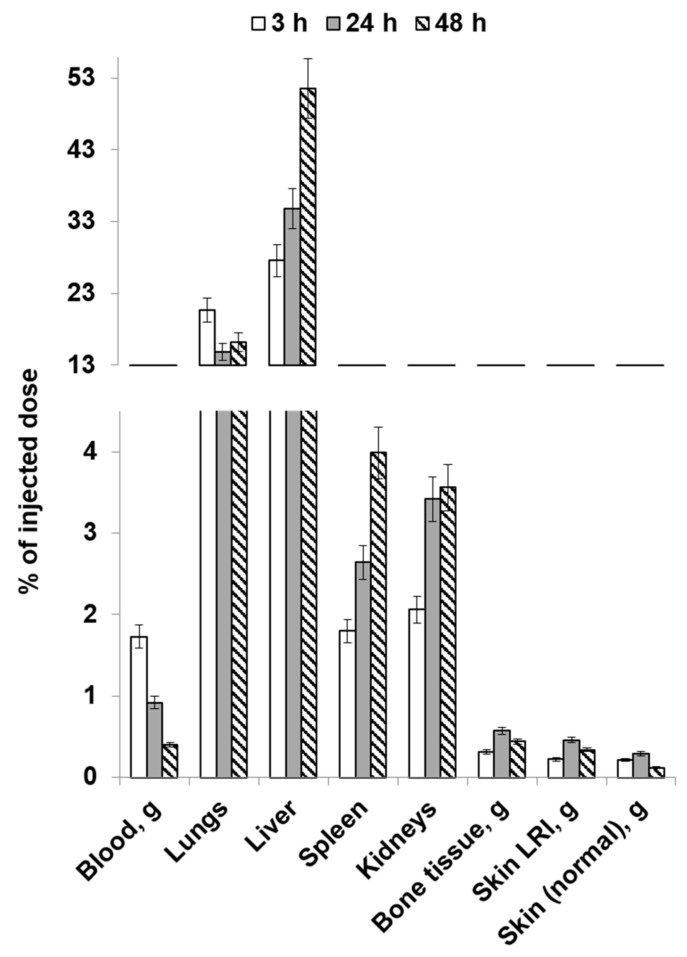
Average values of the distribution dynamics of [^89^Zr]Zr-oxine-labeled MSCs in rats on post-irradiation day 14 (intravenous injection).

Figure 6 and Figure 7 list the average values for the results obtained in the study focusing on the distribution dynamics of human mucosal tissue MSCs labeled with [^89^Zr]Zr-oxine in the body of Wistar rats on post-irradiation day 14 (using local and intravenous routes of administration).

With local administration, a significant number of the injected cells labeled with [^89^Zr]Zr-oxine remained in the skin LRI. Substantial amounts of labeled MSCs were also accumulated in the lungs, liver, and kidneys. The accumulation in these organs decreased by 24 h, and high accumulation levels were observed again by 48 h. The dynamics of activity accumulation in the bone tissue showed that zirconium-89 was released from the cells during the first 3 h after cell introduction. This process almost did not occur the next day, thus indicating that the viability of MSCs in the animals’ bodies was preserved. Emergence of a free radionuclide observed again 48 h post-injection indicates that some of the cells had also lost their viability.

The contrast between the accumulation of labeled MSCs in the local radiation injury focus and in intact skin is even more pronounced than in the animals one day after the exposure. This fact suggests that the development of an inflammatory response in the LRI area contributes to strong retention of MSCs; therefore:

(a) the LRI focus can be clearly visualized using PET, and
(b) effective therapy through the local administration of cells is possible.

For intravenous injection, a very high level of accumulation in the liver was observed across all the time points. In smaller but significant quantities, labeled MSCs were accumulated in the lungs and spleen. The accumulation of cells in the LRI and a healthy skin area did not differ significantly at 3 or 24 h post-injection. By 48 h, the LRI/skin ratio was 3.14 ± 1.17. Accumulation in the kidneys and bone tissue was negligible. Like in the case of the study one day post-irradiation, administration of MSCs via intravenous injection will make it possible to visualize the local radiation injury focus, but not in a direct projection above the liver and lungs. Local injection of MSCs into the visualized focus area is recommended for performing cell therapy.

### 2.5. Study of the Distribution Dynamics of Mesenchymal Stromal Cells Labeled with [^89^Zr]Zr-Oxine in Wistar Rats with Chronic Radiation-Induced Ulcers

**Figure 8:**
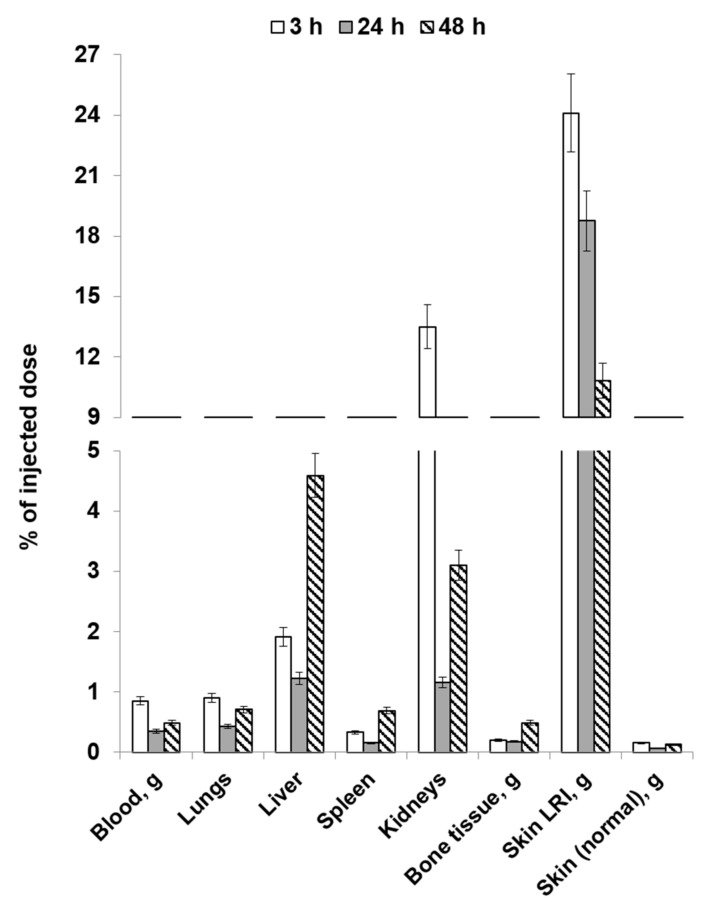
Average values of the dynamics of the distribution of [^89^Zr]Zr-oxine-labeled MSCs in the bodies of rats with chronic radiation-induced ulcers after local injection.

**Figure 9:**
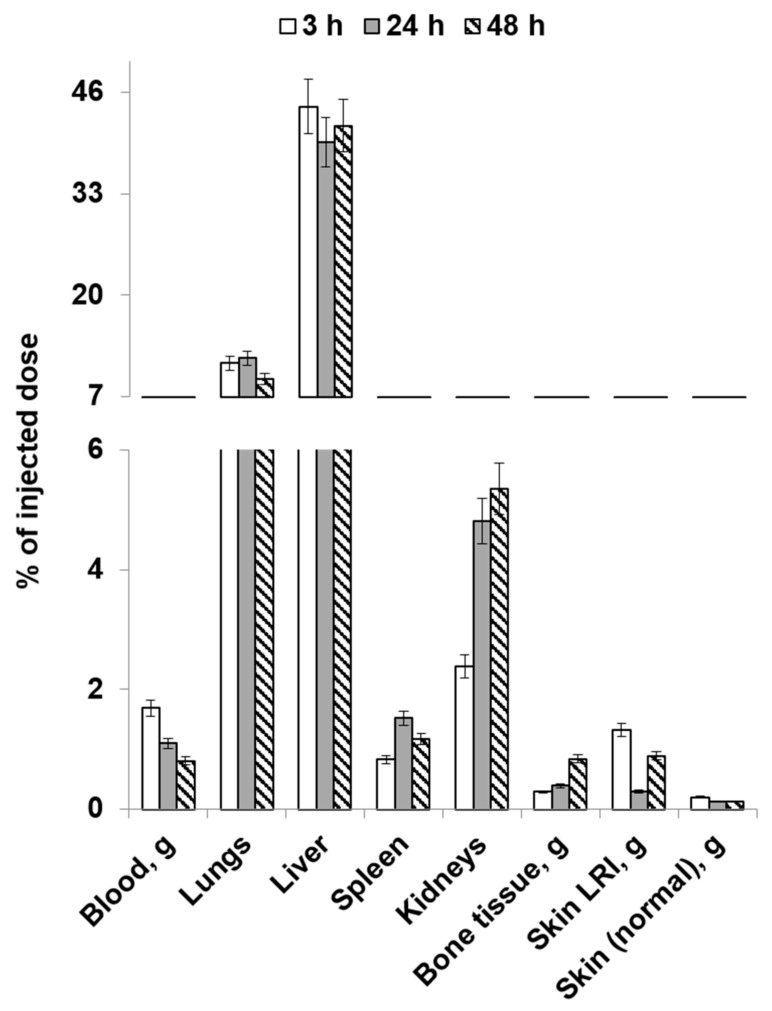
Average values of the dynamics of the distribution of [^89^Zr]Zr-oxine labeled MSCs in the bodies of rats with chronic radiation-induced ulcers after intravenous injection.

Figure 8 and Figure 9 list the average values of the findings obtained in the study, focusing on the distribution dynamics of human mucosal tissue MSCs labeled with [^89^Zr]Zr-oxine in the body of Wistar rats with chronic radiation-induced ulcers after local and intravenous administration.

In the case of local administration, a high level of activity accumulation in the kidneys was observed 3 h after the administration of labeled MSCs, which decreased by 48 h, although it remained higher compared to other organs. Such dynamics indicate that free zirconium-89 was intensively released during the first 3 h after the injection of labeled cells into the body, possibly due to their death.

Activity accumulation in the liver was also observed 48 h after injection. This may be indicative of the migration of MSCs into the vascular bed. A significant number of injected cells remained in the skin LRI, although in smaller amounts than in animals one and 14 days after irradiation. However, chronic ulcers were clearly visible in PET images for up to 48 h.

A high level of accumulation in the liver after intravenous administration was observed across all the time points. The accumulation of free zirconium-89 was detected in the kidneys, which increased from the instant of administration of labeled MSCs and was 5.35 ± 0.99% by 48 h.

In the spleen, the accumulation of labeled MSCs increased to 1.17 ± 0.63% by 48 h post-injection. Conversely, the accumulation in the lungs decreased during the distribution period, which is a physiological process that is also observed for labeled leukocytes. Accumulation in healthy skin and LRI was insignificant and did not differ significantly. This fact indicates that there is a high probability of obtaining doubtful or false positive PET scan results.

## 3. Discussion

Skin LRI is a serious medical, social, and economic problem. It is known that the early effects of skin damage induced by ionizing radiation (dry and wet dermatitis) are associated with damage to the epidermis, while the late effects (skin atrophy, radiation necrosis, etc.) result from damage to the dermis. Thus, the highly radiosensitive cells of the basal layer are the radiation target of the epidermis, while microvasculature vessels are the radiation target of the dermis. As a result, in the case of deep radiation burns, necrotic and degenerative processes cover all skin layers, gradually spreading to deeper tissues and up to the bones. An analysis of the literature data demonstrates the crucial role played by regenerative medicine and cell technologies in treatment of LRI. However, no experimental data on the intravital migration of MSCs when used in local radiation injuries are currently available.

In order to understand the effectiveness of using MSCs, one needs to study their migration after transplantation using a method that would be highly sensitive and specific for individual cell detection and allow for quantitative determination of cells in any anatomical location with the feasibility of monitoring with a high spatial and temporal resolution. In order to efficiently use MSCs, one needs to understand the following important issues: whether cells retain their efficiency and how long they persist in tissues after transplantation, and what route and dose of administration will ensure the successful delivery of MSCs.

Currently, there are very few studies focusing on MSC migration pathways depending on their method of administration in pathological conditions. Studies involving intravenous administration of MSCs labeled with indium-111 oxine in a model of myocardial infarction in dogs [25] showed a high distribution immediately in the lungs post-infusion, followed by a reduction of the number of MSCs and a later redistribution from day 1 to day 7 in various tissues, mainly the liver, spleen, and kidneys. A similar model of myocardial infarction in mice (PCR assessment) [26] showed a smaller number of cells migrating to the lungs compared to other organs (less than 1%). Intravenous administration of MSCs labeled with green fluorescent protein in baboons [27] and evaluation of their distribution in various tissues demonstrated the distribution of MSCs over a long period of time. Tissues of the gastrointestinal tract, kidneys, skin, lungs, thymus, and liver contained MSCs. Similar results were obtained in other studies [28,29,30,31,32]. The redistribution of MSCs can be explained by the fact that, for example, monocytes can change their immunophenotype by inducing Treg cells [33]. Under pathological conditions in certain tissues of an organ, the nature of the distribution of MSCs differed from that in healthy animals. High levels of distribution of MSCs labeled with superparamagnetic iron oxide nanoparticles (SPIONs) were observed in the kidneys of rabbits with acute kidney injury [34], in the intestine [35,36], in the brains of sick animals in a model of Alzheimer’s disease [37,38], with brain tumors in mice [39], in rats and beagles with spinal cord injuries [40,41], in rats with intracerebral hemorrhage [42], and in rats with acute respiratory distress syndrome or liver tumors [43,44]. However, in an experimental mouse model of autoimmune encephalomyelitis [45], MSCs labeled with a fluorescent label were not distributed in the brain. After intravenous administration of placental MSCs labeled with fluorescent nanodiamonds (FNDs), 70% of the cells migrated to the lungs, which was consistent with other studies and with our findings [46,47]. The lungs are the first organ where MSCs are distributed after intravenous injection [48], followed by the liver and kidneys [49]. MSCs are captured in the lungs due to space limitations [50], since MSCs have a diameter of more than ~20 μm and are much larger than the width of lung microcapillaries. After intravenous administration, MSCs labeled with FNDs migrated over time from the lungs to other tissues/organs, such as the liver and spleen, or to the injury site. The number of MSCs labeled with FNDs decreased in the heart and kidneys. In an animal model of induced ischemia–reperfusion injury of the left kidney [46], FND-labeled MSCs injected into the portal vein migrated to the injury site on day 5 by up to 3% compared to the healthy kidneys, where a decrease was noted. The percentage of MSCs labeled with FNDs remained unchanged over time. The percentage of MSCs that migrated to the kidneys was approximately 4%, and the kidneys are likely able to redistribute MSCs *in vivo*.

Assessments of the biodistribution of MSCs labeled with indium-111 oxine after intravenous administration in humans in three studies have been carried out. In a patient with liver cirrhosis, early (up to 48 h) distribution of MSCs was observed in the lungs, with a later decrease and high distribution in the spleen and liver [51]. In a patient with breast cancer, MSCs [52] in the peripheral blood were observed during immunophenotyping, revealing a rapid clearance of MSCs in the blood, while no cells were detected 1 h post-injection. In a patient with hemophilia A [53], MSCs labeled with indium-111 oxine were distributed to the lungs and liver, with a subsequent decrease. Distribution to the bleeding site was noted after 24 h.

Local intradermal injections of luciferase-labeled MSCs in mice [53] demonstrated that MSCs remain in the skin and migrate to the lymph nodes without systemic distribution. In skin wounds [54,55], MSCs (assessed using PCR) are initially distributed diffusely and then concentrated towards the wound edges. There are no data regarding systemic distribution after intradermal injection. There has been only one study of the biodistribution of intranodal injection of MSCs (assessed using PCR), in which most MSCs remained at the injection site or in the adipose tissue around the injected lymph nodes after 48 h [56,57], without systemic distribution of the cells.

We have previously shown that intravenous administration of MSCs labeled with indium-111 in rabbits with acute skin LRI (on day 25) led to their migration from the bloodstream, and they were already recorded in the lungs and kidneys 3 h after injection. Over the next two days, activity in the lungs decreased markedly, while in radiation burns, on the contrary, it increased. However, the activity in the kidneys remained at a high level. With local injection of [^111^In]In-labeled MSCs, it was possible to confirm the almost complete absence of distribution and migration of MSCs from the injection site. Noticeable activity levels were recorded in the kidneys 24 and 48 h after the injection. Injected [^111^In] In-labeled MSCs rapidly migrated into the bloodstream and then accumulated in the LRI, and also noticeably in the kidneys, liver, and lungs [57].

A single study was conducted in humans using local administration of MSCs labeled with iron sucrose (Venofer^®^, Luitpold pharmaceuticals, New York, NY, USA) in the intervertebral discs of four patients [58]. Histological examination after eight months revealed the presence of MSCs in the intervertebral discs with a chondrocyte-like differentiation, and after 28 months, no MSCs were identified in the discs.

There are currently no data on the local administration of [^89^Zr]Zr-oxine-labeled MSCs; there exist only publications on intravenous administration of MSCs labeled with ^89^Zr-oxine in lung cancer in mice [59] (assessed using positron emission tomography–computed tomography). In this study, 1 h after injection, a 60% signal was observed in the lungs, and from 1 to 7 days after administration, there was a decrease in the signal in the lungs to 24.6%. Hence, intravenous administration of MSCs is the simplest and most commonly used form of administration in clinical practice for ensuring cell penetration into organs. Local administration of MSCs, depending on the tissue lesion, can be effective for treating skin lesions when targeted delivery to the affected area is required. The present study has demonstrated that intravenous administration of MSCs from human mucosal tissue labeled with [^89^Zr]Zr-oxine makes it possible to visualize the focus of local radiation injury using PET, and for effective cell therapy, local injection of MSCs into the area of the visualized focus of local radiation injury is recommended.

## 4. Materials and Methods

### 4.1. Animal Groups

The study used 49 laboratory animals (male Wistar rats aged 8–12 weeks weighing 210.0 ± 30.0 g). The rats were procured from the specialized nursery of laboratory animals “Pushchino” (Moscow Region, Russia), had the appropriate veterinary certificate, and were quarantined for 14 days. The laboratory animals were randomized and divided into seven groups (seven animals per group) depending on the therapy type:

Group 1—irradiated rats that received an intradermal injection of [^89^Zr]Zr-oxine-labeled allogeneic MSCs of human mucosal tissue into the ulcerated surface on day 1 after LRI modeling;
Group 2—irradiated rats that received an intradermal injection of [^89^Zr]Zr-oxine-labeled allogeneic MSCs of human mucosal tissue into the ulcerated surface on day 14 after LRI modeling;
Group 3—irradiated rats that received an intradermal injection of [^89^Zr]Zr-oxine-labeled allogeneic MSCs of human mucosal tissue into the ulcerated surface after chronic ulcer formation after LRI modeling (six months);
Group 4—irradiated rats that received intravenous infusion of [^89^Zr]Zr-oxine-labeled allogeneic MSCs of human mucosal tissue on day 1 after LRI modeling;
Group 5—irradiated rats that received intravenous infusion of [^89^Zr]Zr-oxine-labeled allogeneic MSCs of human mucosal tissue on day 14 after LRI modeling;
Group 6—irradiated rats that received intravenous infusion of [^89^Zr]Zr-oxine-labeled allogeneic MSCs of human mucosal tissue after chronic ulcer formation six months after LRI modeling.

Each laboratory animal was examined on days 1 and 2 after LRI simulation. During the examination, the laboratory animals’ conditions were monitored, and their behavior, movements, cardiovascular and/or respiratory functions, changes in appetite and body weight, body temperature, etc., were assessed.

The animals were removed from the experiment on days 1 and 2 after the start.

### 4.2. Modeling of Local Radiation Injuries

Modeling of the relatively “soft” X-ray radiation of LRIs was carried out using an LNK-268 X-ray unit (RAP100-10) (Diagnostics-M, Moscow, Russia) with the following irradiation mode: dose, 110 Gy; a 0.1 mm thick aluminum filter; voltage, 30 kV; beam current, 6.1 mA; and dose rate, 21.1 Gy/min for 312 s (dose accuracy ± 5%, dose measurement uncertainty ± 6%) according to the previously proposed method [19,60,61], leading to a short latency period and chronic skin ulcers in the laboratory animals. After irradiation, the animals were seated in individual sterile boxes with an autonomous smart flow ventilation system (Tecniplast Group, Buguggiate, Italy) with unlimited access to water and food.

### 4.3. Cultivation of Mesenchymal Stromal Cells

Non-personalized human MSCs derived from the gingival mucosa of a healthy patient were taken from the collection of the Cryobank of the Center for Biomedical Technologies Burnasyan Federal Medical Biophysical Center of Federal Medical Biological Agency of Russia, Moscow. The samples were obtained with informed consent and in accordance with the Ethics Committee (Reference number: 85A/20.05.2020). The cryopreserved cells were placed in a water bath at 37 °C for defrosting and cultivation. Cell suspensions were placed in culture flasks (surface area 75 cm^2^) with a concentration of 0.6–0.8 × 10^6^ per flask in a xeno-free medium (Stem Cell, Vancouver, BC, Canada) supplemented with 100 U/mL penicillin, 100 U/mL streptomycin, and 2 mM glutamine from the 3rd to the 5th passage. Culturing was carried out at 37 °C at absolute humidity and 5% CO_2_. The resulting MSCs were administered to the laboratory animals at a calculated dose of 2 × 10^6^ cells per kg.

### 4.4. Immunological Characteristics and Viability of Mesenchymal Stromal Cells

The immunophenotype of MSCs in the human mucosal tissue was determined using flow cytometry. The expression of cell surface markers was assessed using fluorochrome-labeled antibodies against CD34, CD45, CD90, CD105, CD73, and HLA-DR (BD Biosciences and Beckman Coulter, San Jose, CA, USA) and a FACSCanto II flow cytometer (Becton Dickinson, San Jose, CA, USA) according to the manufacturer’s instructions.

Cell viability was assessed using the 7-ADD dye, which penetrates the cytoplasmic cell membrane and binds to its DNA. The number of CD45-negative 7-ADD-positive cells was determined using a FACSCanto II flow cytometer (Becton Dickinson, San Jose, CA, USA) according to the manufacturer’s instructions.

### 4.5. Synthesis of [^89^Zr]Zr-Oxine

Zirconium-89 as [^89^Zr]ZrCl_4_ in 5M HCl with a radionuclide purity >99.9% was purchased from Cyclotron Ltd. (Obninsk, Russia). [^89^Zr]Zr-oxalate was prepared using ZR hydroxamate resin (Triskem International, Bruz, France) and Chelex-100 (Sigma-Aldrich, St. Louis, MO, USA) in a 0.115 M sodium oxalate (рН 4 ± 0.5) solution according to a previously described method [62].

A total of 250 µL of 8-hydroxyoxynaline solution (Sigma-Aldrich) at a concentration of 2 mg/mL in 0.05 M HCl was added to 100 µL of [^89^Zr]Zr-oxalate solution (250 MBq/mL). The final pH was achieved by adding the desired amount of NaOH solution (final volume: 1 mL). The reaction mixture was incubated for 1–24 h at room temperature. Thin-layer chromatography (TLC) and liquid–liquid extraction (LLE) were used for an analysis of radiochemical purity.

For TLC, a silica gel on glass microfiber (iTLC-SG, Agilent, Santa Clara, CA, USA) was used, and ethyl acetate was used as a solvent. In this system, [^89^Zr]Zr-oxalate and ^89^Zr-colloid are characterized by Rf = 0, and the [^89^Zr]Zr-oxine—Rf = 0.9–1 [21].

For the LLE method, a 50 μL aliquot of the [^89^Zr]Zr-oxine preparation was added to a mixture containing 950 μL of saline and 1000 μL of chloroform. The vials were vortexed vigorously for 5 min and stored (5 min) for phase separation. The radioactivity concentration in 50 µL aliquots of each layer (performed in triplicates) was measured using a WIZARD2 automatic γ-counter (PerkinElmer, Waltham, MA, USA). The extraction efficacy was calculated by expressing the radioactivity, found in the organic phase, as a percentage of the total radioactivity due to zirconium-89 measured in both phases.

### 4.6. Radiolabeling of Mesenchymal Stromal Cells

[^89^Zr]Zr-oxine preparations obtained at pH 7–9 and an activity of at least 0.14 ± 0.09 MBq/mL were used for labeling. [^89^Zr]Zr-oxine (100 µL) was added to the prepared cell suspension in 1 mL of saline (0.7 ± 0.2 × 10^6^ MSC). The cells were incubated for 30 min at 37 °C. At the end of the incubation procedure, the labeled cells were pelleted through centrifugation (150 g, 5 min). The supernatant containing unbound [^89^Zr]Zr-oxine was quantitatively removed and transferred to a specially marked tube for radiometry. The remaining cells were carefully resuspended in 1 mL of saline. 

The labeling efficiency (*LE*) of the cells was determined through direct radiometry of the cell suspension and supernatant according to Formula (1):

(1) $$LE=\frac{A_{c}}{A_{c}+A_{s}}\times 100\%,$$

where *A_c_* is the MSC suspension activity (cpm) and *A_s_* is the supernatant activity (cpm). The retention of ^89^Zr (efflux) was estimated in saline through direct radiometry of the cell suspension and supernatant according to Formula (1).

### 4.7. Injection of Radiolabeled Mesenchymal Stromal Cells in the Animals

The following injection routes were used: intravenous (the tail vein) and local (by intradermal puncture of the affected area).

The conditions of the laboratory animals were monitored by assessing of their behavior, movements, cardiovascular and/or respiratory functions, changes in appetite and weight, body temperature, etc. On the follow-up days, their skin surface was examined, and the course of the wound process was assessed (skin damage depth and dimensions (length and width), the total area of the damaged skin, the area of the open wound surface, the presence of discharge, blisters, scabs, desquamated epidermis, color of the exposed dermis, and fibrin plaque).

After the injection of the labeled cells (3, 24, and 48 h post-injection), the animals were euthanized using a partial decapitation method.

### 4.8. Radiometry

The following organs and tissues were taken for radiometry: blood, lungs, liver, spleen, kidneys, femur, heart, LRI skin, and healthy skin. Sample radiometry was carried out using a Wizard 2480 automatic gamma counter (PerkinElmer, Shelton, CT, USA). The count of [^89^Zr]Zr-oxine-labeled cells was expressed as the percentage of the total activity recorded in the animal for the entire organ under study (%/organ), and for samples of blood, skin, local radiation injury, muscle, and bone tissues per g of tissue (%/g). 

The proportion of accumulation of the [^89^Zr]Zr-oxine labeled cells in all circulating blood, skeletal muscles, and skin was calculated using Formulas (2)–(4). The coefficients for recalculating the weights of these tissues and organs from the animal’s body weight were taken from the Reference Manual for Radiobiologists [63]:

(2) $$\%{ID}_{blood}=\frac{A_{blood}}{m_{blood}}\cdot m_{rat}\cdot 0.07\cdot 100\%,$$

where %*ID_blood_* is the proportion of drug accumulation in the blood from the administered activity (%); *A_blood_* is the blood sample count rate (cpm); *m_blood_* is the weight of the blood sample (g); *m_rat_* is the animal’s weight (g); and 0.07 is the blood conversion factor.

(3) $$\%{ID}_{muscle}=\frac{A_{muscle}}{m_{muscle}}\cdot m_{rat}\cdot 0.45\cdot 100\%,$$

where %*ID_muscle_* is the proportion of drug accumulation in muscle tissue from the administered activity (%); *A_muscle_* is the counting rate of the sample with the muscle tissue (cpm); *m_muscle_* is the weight of the muscle tissue sample (g); *m_rat_* is the animal’s weight (g); and 0.45 is the conversion factor for muscle tissue.

(4) $$\%{ID}_{skin}=\frac{A_{skin}}{m_{skin}}\cdot m_{rat}\cdot 0.13\cdot 100\%,$$

where %*ID_skin_* is the proportion of drug accumulation in the skin from the administered activity (%); *A_skin_* is the skin sample count rate (cpm); *m_skin_* is the skin sample weight (g); *m_rat_* is the animal’s weight (g); and 0.13 is the skin conversion factor.

The proportion of the accumulation of %*ID_i_*-labeled cells in the organs and tissues was determined using Formula (5):

(5) $${\%ID}_{i}=\frac{A_{i}}{\sum A_{org}-A_{IP}}\cdot 100\%,$$

where *A_i_* is the organ or tissue sample count rate (cpm); *∑A_org_* is the total score in all the organs and tissues (cpm); and *A_IP_* is the counting rate at the injection site (tail vein; for other types of injection *A_IP_* = 0).

### 4.9. Statistical Analysis

Statistical analyses of the preclinical study data were carried out using adequate mathematical and statistical data processing methods. The choice of procedures and methods for statistical data analysis was based on the study design, its purpose, and selected indicators. All the quantitative data were subjected to mathematical analysis; the average values, standard deviation, standard error of the arithmetic mean, and the confidence interval were calculated. The significance of differences between the compared values was assessed using unpaired two-tailed *t*-tests. Differences were considered significant at a significance level equal to or less than 5% (*p* ≤ 0.05). 

## 5. Conclusions

The biodistribution of [^89^Zr]Zr-oxine-labeled human MSCs is completely safe, as intravenous injection leads to initial cell accumulation in the lungs, with their subsequent redistribution to the liver, spleen, and kidneys. When locally injected into cell tissues, MSCs are not distributed systemically in significant amounts. In this case, the optimal conditions for labeling cells with [^89^Zr]Zr-oxine should be specified individually for each method of introducing MSCs. Intravenous injection of [^89^Zr]Zr-oxine-labeled human MSCs makes it possible to study the migration of MSCs and visualize the focus of local radiation injury using PET. Local injection into the area of local radiation injury makes effective therapy possible.
